# Enhancing Efficiency with an AI-Augmented Clinician in Neurology

**DOI:** 10.14336/AD.2024.1249

**Published:** 2025-10-31

**Authors:** Krish Kapadia, Sanskriti Ruwali, Tanvi Malav, Sridhar Seshadri, Abraham Seidmann, Daniel Z. Press, Vijaya B. Kolachalama

**Affiliations:** ^1^Department of Medicine, Boston University Chobanian & Avedisian School of Medicine, Boston, MA 02118, USA.; ^2^Gies College of Business and Carle Illinois College of Medicine, University of Illinois at Urbana Champaign, Champaign, IL 61820, USA.; ^3^Questrom School of Business, Boston University, Boston, MA 02215, USA.; ^4^Division of Cognitive Neurology, Beth Israel Deaconess Medical Center, Boston, MA 02215, USA.; ^5^Department of Computer Science, Boston University, Boston, MA 02215, USA.; ^6^Faculty of Computing & Data Sciences, Boston University, Boston, MA 02215, USA.

**Keywords:** neurologist shortage, artificial intelligence, AI-augmentation

## Abstract

Integrating artificial intelligence (AI) technologies into neurology promises increased patient access, engagement, and quality of care, as well as improved quality of work life for clinicians. While most studies have focused on comparing AI models to expert performance, we argue for a more practical approach: demonstrating how AI can augment clinical practice. This article presents a framework for pragmatic AI augmentation, addressing the shortage in neurology practices, exploring the potential of AI in opportunistic screening, and encouraging the concept of AI serving as a “co-pilot” in neurology. We discuss recommendations for future studies designed to emphasize human-computer collaboration, ensuring AI enhances rather than replaces clinical expertise.

## Introduction

Technological advances have continually penetrated healthcare, as new tools and innovations are integrated into clinical practice to improve efficiency. An important step to adopting any new technology involves a thorough review by domain experts to vet its suitability before implementation in patient care. This rigorous review process is equally important for AI-based technologies, ensuring they meet the necessary standards and requirements. When AI technologies are integrated into healthcare systems, they can enhance efficiency, leading to what can be termed “augmented intelligence” [1-3]. In neurology, AI-augmentation involves a pragmatic approach where frontline neurologists, geriatricians, psychiatrists, neuropsychologists, epileptologists, neurosurgeons, and neurooncologists assist with the integration of AI tools.

Many articles explore the potential of AI in healthcare to streamline administrative and clinical tasks, thereby allowing healthcare providers to devote more time to patient care. Commonly cited examples include AI's role in automating the triage process and scheduling appointments [4]. Additionally, AI can enhance the management of medical records, such as generating notes based on ambient listening, queuing orders and prescriptions, suggesting diagnoses, and autonomously summarizing parts of the medical record [5]. Moreover, AI-powered chatbots and virtual assistants are being proposed to engage patients by providing timely information and support, potentially alleviating the workload on clinicians [6]. AI-driven analyses of complex patient data are also highlighted for their ability to assist clinicians in making more accurate diagnoses. While these claims are well-supported by research, they often fail to resonate with many clinicians. To gain better acceptance, it is crucial to directly address the practical needs and concerns of frontline healthcare providers. Focusing on the synergistic benefits of AI and human collaboration can help clinicians better appreciate the added value AI brings to their practice.

An article from the Harvard Business Review argued that successful AI implementation hinges on fostering trust and collaboration between humans and AI [7]. They outlined a four-phase approach for integrating AI into the workplace: starting as assistant handling simple tasks, evolving into a monitor providing real-time feedback, becoming a coach offering personalized performance insights, and eventually acting as a teammate in a distributed human-AI cognitive system. Recently, Microsoft introduced *copilot*, a software capable of completing code as you write and even generating code blocks from task descriptions provided by users within their coding applications. Initially, copilot appeared intimidating to software engineers, who feared it might eventually replace their jobs [8]. Concerns also arose regarding the quality and readability of the code it produced. Despite these concerns, copilot has achieved dramatic success, gaining over 1 million users within just six months. A significant factor in its success was framing it as a "copilot": it never assumes the role of the primary coder and only assists when help is requested. This approach alleviated fears of job displacement and distrust, positioning the software as an augmentation tool that is now widely adopted and used by software engineers. This framing can be similarly applied in healthcare. Multiple studies have shown that AI assistance can significantly improve clinical performance compared to clinicians working alone, such as in the context of breast cancer [9-11], fracture detection [12, 13], and other conditions [14]. In the context of neurology, a recent article highlighted how AI can assist movement disorder specialists in diagnosing and managing Parkinson's disease through advanced imaging analysis, personalized prognostic models, wearable sensors, telemedicine, and selection of candidates for deep brain stimulation [15]. In line with this theme, we highlight AI as a tool that enhances rather than replaces neurologist expertise when integrated into everyday clinical activities.

## AI’s role in addressing the neurologist shortage

The shortage of neurologists and geriatricians is a pressing issue globally [16-20], exacerbated by aging populations and the increasing prevalence of diseases such as dementia, Parkinson's disease, and stroke. According to recent estimates, the gap between the demand for neurologists and the available supply is widening [21]. This shortage leads to increased patient wait times, reduced access to specialized care, and increased workload for existing neurologists, potentially affecting the quality of care provided. AI has the potential to mitigate some of the effects of the neurologist shortage in a few ways. It can improve efficiency by automating routine and administrative tasks, allowing practitioners to focus more on patient care, rather than handling scheduling, patient data entry, and preliminary diagnostic processes. AI-powered diagnostic tools can assist neurologists in arriving at a data-driven diagnosis faster, which is particularly useful for identifying conditions such as dementia at an earlier stage, allowing for timely interventions. Additionally, AI-enabled telemedicine platforms can extend the reach of neurologists to underserved areas, while also facilitating remote monitoring of patients with chronic conditions, thereby reducing the need for frequent in-person visits. Moreover, AI can enhance training and education programs by providing virtual simulations and real-time feedback to improve learning and competency [22, 23]. While AI may not solve all challenges in neurology, positioning it as an augmented assistant, much like Microsoft’s *copilot*, to support existing providers can help address the increasing demand for neurological care, particularly for aging populations.

## AI’s role in opportunistic screening of neurological conditions

Integrating AI-enabled opportunistic screening into neurology practices paves the way for enhanced clinical capabilities and improved patient outcomes. Machine learning algorithms, with their advanced pattern recognition capabilities, can analyze medical images to identify subtle abnormalities that might be missed by human experts. This is particularly relevant in neuroradiology, where AI can be applied to explore various conditions, including dementia, stroke, and cancer. For instance, AI models can be trained to detect early signs of neurodegeneration from routine brain MRIs or CT scans. By analyzing vast amounts of imaging data, these models can identify biomarkers and structural changes in the brain that correlate with the onset of these diseases, potentially years before symptoms manifest. AI directed screening in the setting of neurology could highlight individuals who may benefit from earlier diagnosis, leading to earlier treatment, and ultimately better outcomes.

AI's role in managing stroke patients further exemplifies its potential. By swiftly analyzing imaging data to assess the extent of brain damage, AI can expedite decision-making for treatments such as thrombolysis or thrombectomy. This rapid analysis not only saves critical time but also enhances the accuracy of treatment decisions. A recent retrospective study compared time to treatment, number of patients receiving thrombolysis, and overall outcome between two equal-sized cohorts at a university stroke center before and after the adoption of an “e-Stroke Suite” [24]. This software package automates the process of detecting ischemia on non-contrast CT, and occlusions on CT angiography. While other operating procedures remained the same, the findings suggest that adding this AI tool to the radiologist’s repertoire marginally decreased time to treatment and increased number of people receiving thrombolysis; the consensus was that the adoption of the AI software also “increased confidence and speed of image interpretation.”

Beyond neuroradiology, there is significant potential for AI frameworks integrated within electronic health record systems to sift through multimodal patient data and identify abnormal conditions that may have been overlooked or not reported by doctors in their notes. For example, AI can detect the possibility of Parkinson’s disease based on various clinical symptoms, including those noted by other physicians, such as constipation, anosmia, and REM sleep behavior disorder (dream reenactment) [25]. Additionally, AI can aid in diagnosing relatively rare neurological conditions that have distinct imaging characteristics, such as Huntington's disease, cerebral autosomal dominant arteriopathy with subcortical infarcts and leukoencephalopathy (CADASIL), and leukodystrophies. Such capabilities can enhance diagnostic accuracy, support early intervention, and facilitate comprehensive patient management, further demonstrating the value of AI-augmented neurology.

**Figure 1.**
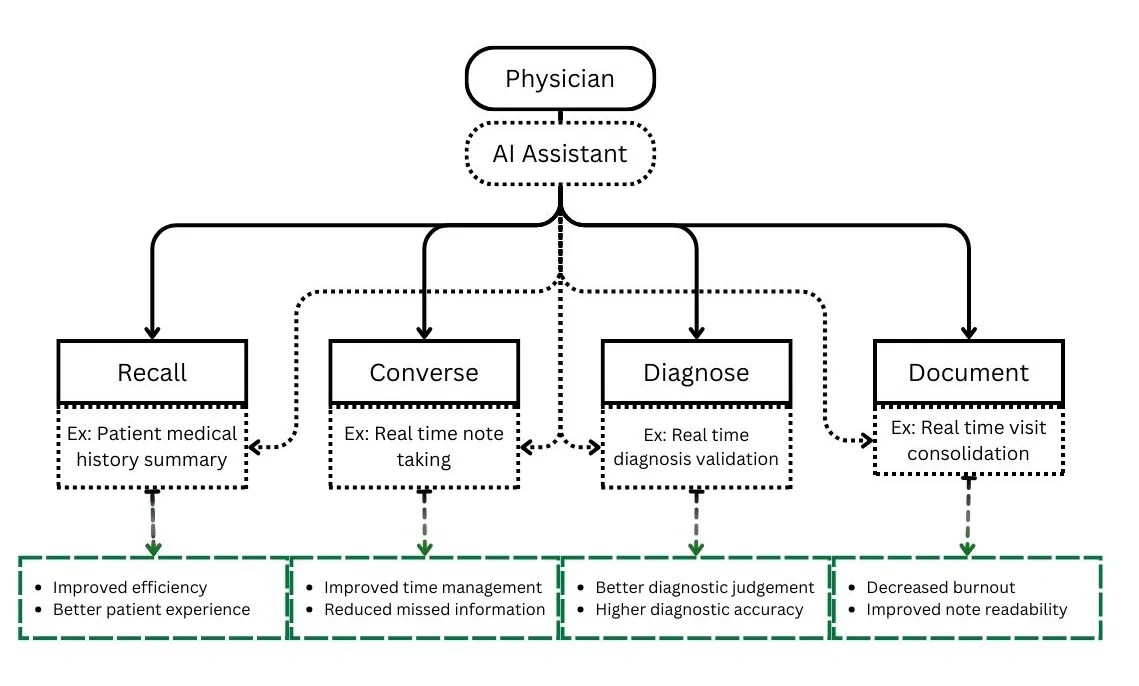
**Integration of an AI assistant in clinical workflows**. The figure illustrates how an AI assistant supports physicians by enhancing four key tasks: (1) Recall (e.g., summarizing patient medical history), (2) Converse (e.g., assisting with real-time notetaking during consultations), (3) Diagnose (e.g., validating diagnostic decisions in real-time), and (4) Document (e.g., consolidating visit notes in real-time). These enhancements are linked to several outcomes: improved efficiency and patient experience, better time management and reduced information gaps, enhanced diagnostic accuracy, and reduced physician burnout and improved note readability.

## AI scalability

AI software holds promise for scalability across various aspects of healthcare. For instance, recent research from Cedars-Sinai in cardiology demonstrated how AI can reliably detect atrial fibrillation (AF) from patient ECGs. In a prospective study, the same AI algorithm was used to classify patients into high- or low-risk categories for AF [26]. The findings revealed a significantly higher incidence of AF among high-risk patients, who were also more likely to develop related cardiovascular conditions. More generalizable AI can assist physicians in a variety of specialities. For example, a recently published article discusses the Permanente Medical Group’s adoption of a smartphone based ambient AI scribe software [27]. This software was able to differentiate between medically relevant and social parts of conversation while transcribing it into a note, saving physicians precious time in documentation. Furthermore, this AI scribe tool was documented to reduce the usage of EHR outside of 7am to 7pm in a “dose-response” way such that physicians who used the tool more spent increasingly less time in the EHR. This software hits upon some of the benefits that AI can provide as it does away with documentation tasks and allows physicians to focus more of their attention on the patient during these short visits. Part of its success can be attributed to how well the platform handles shortcomings: easy integration into workflow, well-established training, and addressing a common clinician complaint without threatening to replace their expertise.

When taking a broader view of an outpatient physician's routine tasks (Fig. 1), the potential scalability of AI becomes evident. However, the current challenge lies in the focus of developing specialized AI tools, where individual tasks such as diagnosis, note-taking, and patient monitoring, are handled by separate software solutions. Each of these tools requires onboarding, training, troubleshooting, and validation, creating a significant barrier to seamless AI integration in healthcare. Nonetheless, there is a growing trend towards more integrated AI systems that combine multiple focused models into a unified interface, like the approach taken by ChatGPT. This type of integration could reduce the burden on physicians by streamlining workflows and simplifying the adoption of AI software. As AI continues to evolve and scale, it could enhance most aspects of clinical practice, leading to measurable improvements in efficiency, accuracy, and patient care.

## Considerations for ongoing and future AI-based studies

The integration of AI into neurology presents some challenges, each with potential solutions. These challenges include physicians misusing the software, applying it for unintended purposes, and uncovering flaws during testing. One possible solution is to adopt practices utilized by medical device companies such as having a company representative available during the initial implementation of a tool to assist clinicians in resolving technical difficulties in real time. This type of support can help ease the adoption process. Similarly, implementation of AI tools into neurology practice can be initiated via pilot studies in real-world environments, much like what was performed for the ambient AI scribe. These pilot programs can serve as a valuable testing ground to identify potential challenges, workflow disruptions, and areas for improvement before the technology is rolled out on a larger scale.

Significant disruptions to established workflows, such as an initial reduction in the number of patients seen possibly due to the extra time required to integrate AI into daily practice, can diminish the perceived benefits of these tools. This problem often stems from the fact that AI software is sometimes developed without sufficient input from healthcare professionals, leading to systems that are difficult to implement seamlessly. To address this in the short term, work practices may need to be adapted as clinicians adjust their workflows to incorporate AI. This adaptation can be supported through comprehensive training, along with the development of clear guidelines and protocols to help ease the transition. Looking ahead, AI tools for healthcare should involve medical professionals throughout the development process to ensure their practical needs are addressed. Starting integration efforts early in software development and progressing incrementally can help reduce workflow disruptions. Continuous feedback mechanisms should be established to monitor the performance of AI tools and their effects on clinical practice. Regular input from clinicians will be vital in refining these tools and improving their utility. Collaboration with IT departments and administrative teams will also be necessary to tackle challenges such as data integration, system compatibility, and training, ensuring that AI is successfully integrated into clinical settings.

Concerns about beliefs and identity may arise, with some providers fearing that AI will replace their expertise. Emphasizing AI as a tool to augment and enhance human expertise and highlighting success stories where AI has improved clinical outcomes and reduced clinician workload, can help mitigate these fears. For example, a recent study demonstrated that a multimodal AI model utilizing routinely collected clinical data improved differential diagnosis of dementia [28]. In a randomly selected subset of 100 cases, the area under the receiver operating characteristic (AUROC) curve for neurologist-led assessments on the diagnostic task, when augmented by the AI model, exceeded neurologist-only evaluations by over 26%. In another study, a deep-learning model for detecting brain metastases on 3D post-contrast MRI not only improved detection sensitivity to 95.8% but also significantly reduced radiologists' reading time by 47% for trainees and 32% for experienced radiologists [29]. Including metrics such as reading time and other work-life factors in addition to accuracy improvements may encourage AI adoption by physicians.

The issues of data quality and access become critical, as AI systems rely on high-quality data for accurate predictions and recommendations. Implementing standardized data collection and management practices and collaborating with technology developers to create robust AI systems, can ensure the effectiveness of AI tools. Furthermore, ethical and regulatory concerns must be addressed, particularly regarding patient privacy, data security, and informed consent [30-33]. The U.S. Food and Drug Administration (FDA) has adopted a distinctive approach to regulating AI. Instead of attempting to control the continuous evolution of AI technologies, the FDA focuses on establishing rigorous standards for privacy, data security, and consent at the company level. As the AI landscape evolves, new measures are being developed to ensure ongoing patient consent throughout the process as well as work towards integrating these safeguards into existing regulatory frameworks.

To clearly identify the value that an AI-based system can bring to healthcare, we need to perform studies, both retrospective and prospective, involving frontline providers in neurology. A comprehensive result would include a set of performance measures, such as diagnostic accuracy, time saved, and patient-related outcomes, comparing scenarios where providers work alone versus when they are assisted by AI technology. This comparison can highlight added value in the form of time saved, increased revenue, improved accuracy, and better outcomes. Training programs for clinicians should be established to enhance their proficiency in using AI tools. These programs can include simulation-based learning modules where clinicians can practice using AI tools in a controlled environment. Simulations can help clinicians become familiar with AI interfaces, understand their functionalities, and learn how to interpret AI-generated insights effectively. One could also establish continuous professional development opportunities to keep clinicians updated on the latest advancements in AI technology.

It must also be noted that AI is often held to higher standards of performance and explainability compared to human experts, despite both being prone to errors and biases. This higher standard is likely due to the perception that a machine's decisions should be infallible in part due to medico-legal aspects, making comparisons to human performance less relevant. This stringent expectation may delay AI adoption, which can be beneficial in ensuring thorough evaluation. Moreover, the adoption of AI in neurology practices can be slow due to these initial expectations and misaligned financial incentives within the healthcare system. Given these practical challenges, exaggerated claims about AI replacing neurologists or other clinicians could deter medical students from entering the field, while undue opposition to AI could negatively impact patient outcomes. Both AI and human decision-making processes necessitate balanced expectations and rigorous testing. Ultimately, the goal should be to develop neurology practices that benefit patients, leveraging a reasonable level of trust in AI for assistive care while maintaining confidence in human decision-making.

## Conclusion

A pragmatic approach to AI-augmentation that outlines challenges and potential solutions related to the incorporation of AI-based technologies into neurologists' everyday workflow is much needed. We propose framing AI software to be integrated in healthcare settings as copilots or assistive technologies to quell fears that providers and patients may harbor surrounding AI integration in their work or treatment, respectively. Such an approach can help frontline care providers use AI-based technologies to transform how neurological conditions are understood and treated.
